# Diagnostic ability of limited volume cone beam computed tomography with small voxel size in identifying the superior and inferior walls of the mandibular canal

**DOI:** 10.1186/s40729-018-0133-7

**Published:** 2018-07-26

**Authors:** Hiroko Ishii, Akemi Tetsumura, Yoshikazu Nomura, Shin Nakamura, Masako Akiyama, Tohru Kurabayashi

**Affiliations:** 1 Department of Oral and Maxillofacial Radiology, Graduate School of Medical and Dental Sciences, Tokyo Medical and Dental University, 1-5-45, Yushima, Bunkyo-ku, Tokyo, Japan; 2 URA, Research Administration Division, Tokyo Medical and Dental University, 1-5-45, Yushima, Bunkyo-ku, Tokyo, Japan

**Keywords:** CBCT, Mandibular canal, Dental implants

## Abstract

**Background:**

The aim of this study was to evaluate the visibility of the superior and inferior walls of the mandibular canal separately using limited volume cone beam computed tomography (CBCT) with small voxel size.

**Methods:**

CBCT cross-sectional images of 86 patients obtained by 3D Accuitomo FPD and reconstructed with a voxel size of 0.08 mm were used for the evaluation. A 30-mm range of the mandible just distal to the mental foramen was divided into three equal areas (areas 1, 2, and 3, from anterior to posterior). Each area contained 10 cross-sectional images. Two observers evaluated the visibility of the superior and inferior walls of the mandibular canal on each of the cross-sectional images in these three areas. The visibility ratio in each area was determined as the number of cross-sectional images with a visible wall divided by 10.

**Results:**

In all areas, the visibility ratio of the superior wall was significantly lower than that of the inferior wall. As for variance among the three areas, the ratio was highest in the most posterior area (area 3) and tended to decrease gradually towards the mental foramen for both walls. Cases in which more than two thirds of the superior wall could be identified (visibility ratio of 0.7 or more) in areas 1, 2, and 3 were 44, 62, and 66%, respectively.

**Conclusions:**

The superior wall was significantly more poorly visualized than the inferior wall in all areas examined. The visibility of the superior wall on CBCT images was limited even when a limited volume device with small voxel size was used.

## Background

The mandibular canal is an important anatomical structure that contains the neurovascular bundle, i.e., the inferior alveolar nerve and artery. The location of the mandibular canal must be correctly identified prior to dental implant surgery to avoid complications including intraoperative and postoperative hemorrhage and neurosensory loss. Cone beam computed tomography (CBCT) is considered the imaging modality of choice for this purpose [1, 2] and is widely used for dental implant treatment planning. Several studies have evaluated the visibility of the mandibular canal on CBCT images [3–8]. However, the results varied widely, around 50–90%, among the studies. Further, no study has evaluated the superior and inferior walls of the canal separately by CBCT, although the location of the former is more important than that of the latter.

Another issue that should be noted for CBCT is the large variability in spatial resolution among devices. High-resolution devices offer the smallest voxel sizes, as small as 0.08 mm or even less [9, 10]. However, the previous studies all evaluated CBCT images having voxel sizes of 0.2 mm or more [3–8], which does not sufficiently reflect the diagnostic advantage of CBCT in demonstrating fine structures. Thus, further study is necessary to evaluate the diagnostic ability of CBCT in identifying the mandibular canal.

The purpose of our study was to evaluate the visibility of the superior and inferior walls of the mandibular canal using limited-volume CBCT with a small voxel size.

## Methods

This study was approved by an institutional review board of our university (D2016-061).

### Patients

Among the patients whose mandibles were examined by CBCT at our dental hospital between April 2012 and August 2016, 96 patients who fulfilled the following two conditions were selected.

On CBCT imaging:

- The smallest field of view (FOV) of the device, 40 × 40 mm, was used.
- The mental foramen and the mandibular body over a range of 30 mm or more just distal to the foramen were imaged.

Of those, 10 patients were excluded because the mandibular canals were affected by lesions on the images. The remaining 86 patients (31 male and 55 female; mean age, 55 years; age range, 19–79 years) were included in this study. The reasons for the CBCT study were to assess a dental lesion in 56 (periapical lesion in 51, root fracture in 4, and periodontal disease in 1) and treatment planning for dental implants in 30 patients.

### Imaging

CBCT images were obtained using 3D Accuitomo FPD (Morita Corp., Kyoto, Japan) operated at tube voltage of 87–90 kV, tube current of 5–8 mA, and scan time of 9 or 18 s. In all cases, the smallest FOV, 40 × 40 mm, was used and the images were reconstructed with a voxel size of 0.08 mm.

### Evaluation of images

**Fig. 1:**
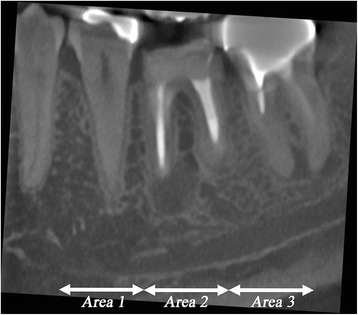
Cross-sectional images in the range of 30 mm just distal to the mental foramen were used for evaluation. The range was divided into three areas, each of which was 10 mm in length, designated as area 1, area 2, and area 3, from anterior to posterior. (The mental foramen was identified on another section and was not visualized on this image)

Using OsiriX software version 3.8.1 (http://www.osirix-viewer.com), cross-sectional CBCT images of the mandible with 1-mm thickness and at 1-mm intervals were reformatted. After the mental foramen was localized, cross-sectional images in a range of 30 mm just distal to the foramen were used for the evaluation. The range was divided into three areas, each of which was 10 mm in length. These were designated as area 1, area 2, and area 3, from anterior to posterior. Each area contained 10 cross-sectional images (Fig. 1).

**Fig. 2:**
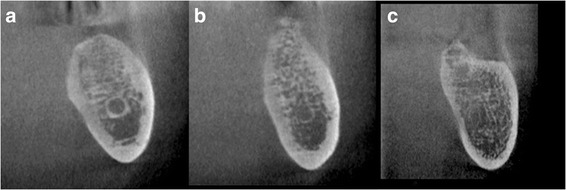
Visibilities of the superior and inferior walls of the mandibular canal. **a** Both walls are visible. **b** Only the inferior wall is visible. **c** Neither of the walls is visible

Two observers (A.T. and H.I., with over 20 years’ and 3 years’ experience as oral radiologists, respectively) independently evaluated the images in a darkened room for the presence or absence of visualization of the superior and inferior walls of the mandibular canal in each of the 10 cross-sectional images in all three areas (Fig. 2). For the purpose of calibration, training was held using typical images prior to the evaluation. Each observer was blind to the other’s results. When disagreement existed between the two observers, another observer (T.K., with over 30 years’ experience as oral radiologist) made a final judgment. After the evaluation, the visibility ratio of the superior and inferior walls in each area was determined as follows:

$$ \mathrm{Visibility}\ \mathrm{ratio}=\mathrm{number}\ \mathrm{of}\ \mathrm{cross}-\mathrm{sectional}\ \mathrm{images}\ \mathrm{with}\ \mathrm{visible}\ \mathrm{wall}/10 $$

The ratio ranged from 0 to 1.

### Sample size

Sample size was determined using the free software G* Power 3.1 [11]. We evaluated 30 patients, and the effect size was calculated from the mean, standard deviation, and correlation. Wilcoxon signed-rank sum test was chosen, and the significance level was set to 0.05. The result showed that a sample size of 26 to 75 patients would provide a power of at least 0.8 for the difference between the superior and inferior walls. For the difference between areas, 27 to 48 patients were needed to provide a power of at least 0.8. Thus, the sample size in our study, 86 patients, was considered sufficient.

### Statistical analysis

Interobserver agreement was evaluated by weighted κ-statistics. A κ-value of 0–0.2 was considered poor agreement, 0.2–0.4 fair agreement, 0.4–0.6 moderate agreement, 0.6–0.8 substantial agreement, and 0.8–1.0 almost perfect agreement [12].

To compare the visibility ratio between the superior and inferior walls in each area, the Wilcoxon signed-rank test was used. Further, to compare the visibility ratio of each wall among the three areas, post hoc comparisons with Scheffe’s test to make multiple comparisons following Friedman’s test were used. Analysis was performed with statistical software, Ekuseru-Toukei 2008, v. 1.10 (Social Survey Research Information Co., Ltd., Tokyo, Japan). A *p* value of < 0.05 was considered statistically significant.

## Results

Interobserver agreement was substantial or almost perfect agreement (Table 1).

**Table 1:** κ-values for interobserver agreement

Mandibular canal wall	Area 1	Area 2	Area 3
Superior wall	0.7795	0.7744	0.7380
Inferior wall	0.8433	0.8815	0.8887

**Fig. 3:**
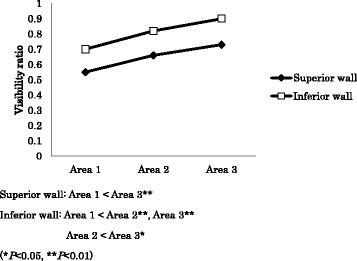
Visibility ratios of the superior and inferior walls in three areas. The Friedman test and Scheffe’s test were used for the statistical analysis

**Fig. 4:**
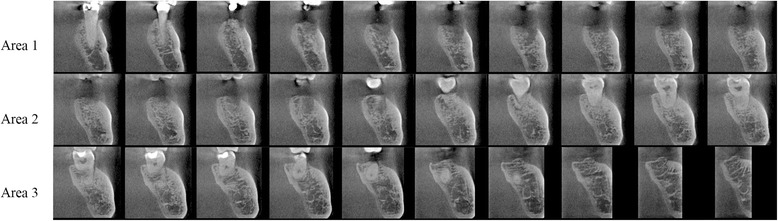
Cross-sectional images of areas 1–3 of a 39-year-old female. The visibility ratios for the superior wall in areas 1, 2, and 3 were 0.2, 0.9, and 0.9, respectively, whereas those of the inferior wall were 0.7, 0.9, and 1.0, respectively

The mean values of the visibility ratio of the superior and inferior walls in each area are shown in Table 2 and Fig. 3. In all areas, the ratio of the superior wall was significantly lower than that of the inferior wall (*p* = 0.0000). As for variance among the three areas, the ratio was highest in the most posterior area (area 3) and tended to decrease gradually towards the mental foramen for both walls. For the superior wall, the ratio of area 1 was significantly lower than that of area 3 (*p* = 0.0006). In contrast, for the inferior wall, significant differences were found between area 1 and area 2 (*p* = 0.0001), area 1 and area 3 (*p* = 0.0000), and area 2 and area 3 (*p* = 0.0132). A representative case is shown in Fig. 4.

**Table 2:**
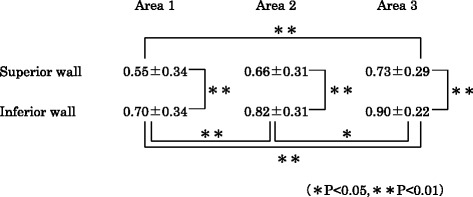
Mean visibility ratio ± SD

Table 3 shows the frequency of cases with visibility ratios of 0.7 or greater (i.e., more than two thirds of the wall was visible) in each area. Cases in which more than two thirds of the superior wall could be identified on CBCT images in areas 1, 2, and 3 were 44, 62 and 66%, respectively.

**Table 3:** Frequency of cases with visibility ratio of 0.7 or more

	Number of cases		
Mandibular canal wall	Area 1	Area 2	Area 3
Superior wall	38 (44%)	53 (62%)	57 (66%)
Inferior wall	57 (66%)	68 (79%)	77 (90%)

## Discussion

It is very important to know the location of the mandibular canal prior to dental implant surgery to avoid surgical complications including vascular trauma or nerve damage.

CBCT is widely accepted to be the imaging method of choice for obtaining this information [1, 2]. However, it is well known that the mandibular canal cannot usually be identified over its entire course even when CBCT is used. Shokri et al. [3] reported that CBCT could demonstrate both sides of the mandibular canal in 87.5% of cases. In contrast, Miles et al. [4] evaluated 360 CBCT cross-sectional images of the premolar and molar regions and reported that the mandibular canal was only visualized in just over half of the images (56%). So, the diagnostic ability of CBCT in identifying the mandibular canal differs widely among studies. Further, localizing the superior wall of the canal is more important than the inferior wall because information about the distance from the former to the alveolar crest is essential for dental implant surgery treatment planning [13]. However, to the best of our knowledge, no study has evaluated visualization of the two walls separately using CBCT.

Another concern regarding CBCT is the large variability in spatial resolution among devices. High-resolution scanners offer the smallest voxel sizes, which are as small as 0.08 mm or even less [9]. Although voxel size may not be identical to spatial resolution, a smaller voxel size generally provides better resolution [9, 10, 14, 15]. However, all of the previous studies that evaluated visibility of the mandibular canal by CBCT used large FOV protocols in which images were reconstructed with voxel sizes of 0.2 mm or greater [3–8]. These studies thus did not sufficiently reflect the diagnostic advantage of CBCT in demonstrating fine structures. In this study, we used a 3D Accuitomo scanner. We selected the smallest FOV (40 × 40 mm) available in this device, providing a voxel size of 0.08 mm. Pauwels et al. [10] compared the spatial resolutions of 13 CBCT and 1 medical CT devices by line pair test and reported that 3D Accuitomo showed the highest resolution. Similarly, Liang et al. [16] compared the visibility of anatomical structures including trabecular bone, periodontal ligament, and lamina dura among five CBCT and one medical CT and concluded that 3D Accuitomo yielded the best results. According to those studies, 3D Accuitomo is one of the best commercially available CBCT devices with regard to image quality. It was thus suitable for the purpose of our study to evaluate the diagnostic ability of CBCT with high spatial resolution in identifying the mandibular canal.

In this study, we only used CBCT images of the mandible obtained with the smallest FOV, 40 × 40 mm. On those images, the range of 30 mm in length in the mandible just posterior to the mental foramen was divided into three equal areas, each of which was 10 mm in length. They were designated as areas 1, 2, and 3, from anterior to posterior. After that, the visibilities of the superior and inferior walls of the mandibular canal in each area were evaluated separately. Although the location of the mental foramen differs among individuals, it is mostly situated below the second premolar or between the apices of the first and second premolars [17, 18]. Thus, it is considered that areas 1, 2, and 3 in our study nearly corresponded to the second premolar to first molar region, the first molar region, and the second molar region, respectively. Visualization of the superior wall in our study was significantly poorer than that of the inferior wall in all areas. Further, concerning the variance among areas, the visibility ratio was highest in the most posterior area (area 3) and tended to decrease gradually towards the mental foramen for both walls.

Although there have been no detailed studies using CBCT, poorer visualization of the superior wall compared with the inferior wall has been reported by some studies using conventional radiographs or medical CT images [19–21]. Whether the wall of the mandibular canal is visible or invisible on images largely depends on the presence or absence of corticalization of the wall surrounding the neurovascular bundle. Bertl et al. [22] performed histomorphological observation of the mandibular canal wall using thin sections of the first molar region of the mandible from 50 cadavers. They identified corticalization of the cranial (superior) and caudal (inferior) wall in 65% and 81%, respectively. Although they only observed the first molar area, their results may be considered consistent with ours of poorer visibility of the superior wall on CBCT images. The presence of nerves and vessels rising to the lower teeth from the mandibular canal may partly explain the lower corticalization rate of the superior wall [23, 24]. Further, the presence or absence of corticalization of the canal wall may be correlated with the trabecular bone volume or density [22, 25]. However, quantitative evaluation of the trabecular bone was difficult in our study using CBCT images. On the other hand, concerning the variance in the visibility of the mandibular canal based on anteroposterior location, several studies using CBCT reported that the mandibular canal can be more easily identified in the posterior region compared with the anterior region [3, 5–8]. An anatomical study using cadavers has reported similar results [23]. Our study evaluated the superior and inferior walls separately, with similar results, although a significant difference was only found between area 3 and area 1 for the superior wall.

Jung and Cho [6] reported that the mandibular canal was clearly visible in 50% of CBCT images in the first molar and in 58% in the second molar region. Similarly, Oliveira-Santos et al. [5] reported that it was visible in 63, 66, and 67% of second premolar, first molar, and second molar regions, respectively. As described above, these studies did not discriminate between the superior and the inferior walls. In our study, cases in which more than two thirds of the superior wall was identified on CBCT images (visibility ratio of 0.7 or more) in areas 1, 2, and 3 were 44, 62, and 66%, respectively (Table 3). It may be difficult to compare the results of our study with those of previous studies because of marked differences of evaluation methods. However, we consider that our results indicate nearly the maximum visibility of the mandibular canal when using CBCT, because we used a limited volume CBCT device with inherent small voxel size, as described above.

Because of poor visualization of the superior wall of the mandibular canal, some ingenuity may be necessary when using CBCT for treatment planning of dental implant surgery. The simplest method is to utilize the average diameter of the mandibular canal. Koivisto et al. [26] evaluated CBCT images and reported that the average diameter of the right and the left mandibular canal in the premolar/molar region was 2.91 and 3.03 mm, respectively. Utilizing these data, the approximate location of the superior wall can be estimated in cases in which the inferior wall was visible. As another method to localize the mandibular canal on CT, the use of panoramic views in addition to cross-sectional views has been recommended [27]. Probably, the imaging modality with the highest visibility of the mandibular canal is high-resolution MRI with small voxel size. Deepho et al. [28] recently reported that 3D-VIBE images at 3T MRI with voxel size of 0.8 mm clearly demonstrated the mandibular canal in 144 out of 147 areas of 62 mandibles. However, MRI has not become a routine imaging technique for dental implant treatment because of its low availability and high cost.

Our study had some limitations that should be addressed. First, in our study, antero-posterior location of the mandibular canal was defined by the distance from the mental foramen. Tooth positions could not be used as a reference, because premolars and molars were totally or partially missing in considerable number of the cases. Although areas 1–3 were considered mostly to correspond to the area from the second premolar to second molar, it might not be true for some cases due to anatomical variations for the position of the mental foramen. Second, we did not evaluate the difference of the visibility of the mandibular canal by age or gender. According to the study by Miles et al. [4], the visibility was significantly lower in females than in males. It was also affected by age depending on the location. Although we applied power analysis to determine the sample size, the sample size was not sufficient for such analysis. Third, we could not confirm the actual positions of the mandibular canal walls because we used CBCT images of clinical cases. Thus, it might be possible that a few cases with misinterpretation were included in our data.

## Conclusions

In conclusion, we evaluated the visibility of the mandibular canal walls on limited volume CBCT images with a small voxel size. Evaluation was performed in the range of 30 mm in length just posterior to the mental foramen, which was divided into three equal areas (areas 1, 2, and 3, from anterior to posterior). The superior wall was significantly more poorly visualized than the inferior wall in all areas. Cases in which more than two thirds of the superior wall was identified on CBCT images in areas 1, 2, and 3 were 44, 62 and 66%, respectively.

## Abbreviations

CBCT: Cone beam computed tomography
FOV: Field of view

## References

[1] Tyndall DA, Price JB, Tetradis S, Ganz SD, Hildebolt C, Scarfe WC (2012). Position statement of the American Academy of Oral and Maxillofacial Radiology on selection criteria for the use of radiology in dental implantology with emphasis on cone beam computed tomography. Oral Surg Oral Med Oral Pathol Oral Radiol.

[2] Weckx A, Agbaje JO, Sun Y, Jacobs R, Politis C (2016). Visualization techniques of the inferior alveolar nerve (IAN): a narrative review. Surg Radiol Anat.

[3] Shokri A, Shakibaei Z, Langaroodi AJ, Safaei M (2014). Evaluation of the mandibular canal visibility on cone-beam computed tomography images of the mandible. J Craniofac Surg.

[4] Miles MS, Parks ET, Eckert GJ, Blanchard SB (2016). Comparative evaluation of mandibular canal visibility on cross-sectional cone-beam CT images: a retrospective study. Dentomaxillofac Radiol.

[5] Oliveira-Santos C, Cappelozza ALÁ, Dezzoti MSG, Fischer CM, Poleti ML, Rubira-bullen IRF (2011). Visibility of the mandibular canal on CBCT crosssectional images. J Appl Oral Sci.

[6] Jung YH, Cho BH (2014). Radiographic evaluation of the course and visibility of the mandibular canal. Imaging Sci Dent.

[7] Angelopoulos C, Thomas S, Hechler S, Parissis N, Hlavacek M (2008). Comparison between digital panoramic radiography and cone-beam computed tomography for the identification of the mandibular canal as part of presurgical dental implant assessment. J Oral Maxillofac Surg.

[8] de Oliveira-Santos C, Souza PH, de Azambuja Berti-Couto S, Stinkens L, Moyaert K, Rubira-Bullen IR, Jacobs R (2012). Assessment of variations of the mandibular canal through cone beam computed tomography. Clin Oral Investig.

[9] Brüllmann D, Schulze RK (2015). Spatial resolution in CBCT machines for dental/maxillofacial applications—what do we know today?. Dentomaxillofac Radiol.

[10] Pauwels R, Beinsberger J, Stamatakis H, Tsiklakis K, Walker A, Bosmans H, Bogaerts R, Jacobs R, Horner K; SEDENTEXCT Project Consortium. Comparison of spatial and contrast resolution for cone-beam computed tomography scanners. Oral Surg Oral Med Oral Pathol Oral Radiol 2012; 114: 127-135. 10.1016/j.oooo.2012.01.020 22727102

[11] Faul F, Erdfelder E, Lang A-G, Buchner A (2007). G*Power 3: a flexible statistical power analysis program for the social, behavioral, and biomedical sciences. Behav Res Methods.

[12] Kundel HL, Polansky M (2003). Measurement of observer agreement. Radiology.

[13] Alhassani AA, AlGhamdi AS (2010). Inferior alveolar nerve injury in implant dentistry: diagnosis, causes, prevention, and management. J Oral Implantol.

[14] Waltrick KB, Nunes de Abreu Junior MJ, Corrêa M, Zastrow MD, Dutra VD (2013). Accuracy of linear measurements and visibility of the mandibular canal of cone-beam computed tomography images with different voxel sizes: an in vitro study. J Periodontol.

[15] Hassan BA, Payam J, Juyanda B, van der Stelt P, Wesselink PR (2012). Influence of scan setting selections on root canal visibility with cone beam CT. Dentomaxillofac Radiol.

[16] Liang X, Jacobs R, Hassan B, Li L, Pauwels R, Corpas L, Souza PC, Martens W, Shahbazian M, Alonso A, Lambrichts I (2010). A comparative evaluation of cone beam computed tomography (CBCT) and multi-slice CT (MSCT) Part I. on subjective image quality. Eur J Radiol.

[17] Greenstein G, Tarnow D (2006). The mental foramen and nerve: clinical and anatomical factors related to dental implant placement: a literature review. J Periodontol.

[18] de Oliveira Júnior MR, Saud AL, Fonseca DR, De-Ary-Pires B, Pires-Neto MA, de Ary-Pires R (2011). Morphometrical analysis of the human mandibular canal: a CT investigation. Surg Radiol Anat.

[19] Denio D, Torabinejad M, Bakland LK (1992). Anatomical relationship of the mandibular canal to its surrounding structures in mature mandibles. J Endod.

[20] Kamrun N, Tetsumura A, Nomura Y, Yamaguchi S, Baba O, Nakamura S, Watanabe H, Kurabayashi T (2013). Visualization of the superior and inferior borders of the mandibular canal: a comparative study using digital panoramic radiographs and cross-sectional computed tomography images. Oral Surg Oral Med Oral Pathol Oral Radiol.

[21] Kubilius M, Kubilius R, Varinauskas V, Žalinkevičius R, Tözüm TF, Juodžbalys G (2016). Descriptive study of mandibular canal visibility: morphometric and densitometric analysis for digital panoramic radiographs. Dentomaxillofac Radiol.

[22] Bertl K, Heimel P, Reich KM, Schwarze UY, Ulm C (2014). A histomorphometric analysis of the nature of the mandibular canal in the anterior molar region. Clin Oral Investig.

[23] Starkie C, Stewart D (1931). The intra-mandibular course of the inferior dental nerve. J Anat.

[24] Carter RB, Keen EN (1971). The intramandibular course of the inferior alveolar nerve. J Anat.

[25] Naitoh M, Katsumata A, Kubota Y, Hayashi M, Ariji E (2009). Relationship between cancellous bone density and mandibular canal depiction. Implant Dent.

[26] Koivisto T, Chiona D, Milroy LL, McClanahan SB, Ahmad M, Bowles WR (2016). Mandibular canal location: cone-beam computed tomography examination. J Endod.

[27] Takahashi A, Watanabe H, Kamiyama Y, Honda E, Sumi Y, Kurabayashi T (2013). Localizing the mandibular canal on dental CT reformatted images: usefulness of panoramic views. Surg Radiol Anat.

[28] Deepho C, Watanabe H, Kotaki S, Sakamoto J, Sumi Y, Kurabayashi T (2017). Utility of fusion volumetric images from computed tomography and magnetic resonance imaging for localizing the mandibular canal. Dentomaxillofac Radiol.

